# The Long Shadow of Early Poverty: Poverty at Birth, Epigenetic Changes at Age 15, And Youth Outcomes at Age 22

**DOI:** 10.31586/crph.2024.990

**Published:** 2024-10-31

**Authors:** Shervin Assari, Mohammad Dezfuli, Amirreza Peyrovinasab, Hossein Zare

**Affiliations:** 1 Department of Internal Medicine, Charles R. Drew University of Medicine and Science, Los Angeles, CA, United States; 2 Department of Family Medicine, Charles R. Drew University of Medicine and Science, Los Angeles, CA, United States; 3 Department of Urban Public Health, Charles R. Drew University of Medicine and Science, Los Angeles, CA, United States; 4 Marginalization-Related Diminished Returns (MDRs) Center, Los Angeles, CA, United States; 5 Molecular Parasitology Laboratory, Pasteur Institute of Iran, Tehran, Iran; 6 Tehran Medical Sciences, Islamic Azad University, Tehran, Iran; 7 Department of Health Policy and Management, Johns Hopkins Bloomberg School of Public Health, Baltimore, MD, United States; 8 School of Business, University of Maryland Global Campus (UMGC), Adelphi, MD, United States

**Keywords:** Race/Ethnicity, Poverty, Epigenetic Aging, GrimAge, School Discipline, Youth, Structural Equation Modeling (SEM), Health Disparities, Longitudinal Study, Biological Embedding, Early Life Adversity

## Abstract

**Background:**

Early life socioeconomic conditions and race/ethnicity are critical determinants of long-term health and behavioral outcomes. Epigenetic changes, particularly those measured by the GrimAge biomarker, may mediate the impact of these early adversities on later life outcomes. This study investigates the relationships between race/ethnicity, poverty at birth, epigenetic aging at age 15, and subsequent self-rated health, school discipline, depression, and school dropout at age 22. We explored sex differences in these paths.

**Methods:**

Data were drawn from the Fragile Families and Child Wellbeing Study (FFCWS), which included 733 youth with comprehensive follow-up data up to age 22. Structural Equation Modeling (SEM) was employed to assess the pathways from race/ethnicity and poverty at birth to epigenetic aging (GrimAge) at age 15, and subsequently to self-rated health and school discipline at age 22. The model controlled for potential confounders including sex, family structure, and parental education.

**Results:**

Race/ethnicity and poverty at birth were significantly associated with higher GrimAge scores at age 15 (p < 0.05). Higher GrimAge scores were predictive of poorer self-rated health (β = −0.08, p < 0.05) and increased instances of school discipline (β = 0.13, p < 0.01) at age 22. The indirect effects of race/ethnicity and poverty at birth on self-rated health and school discipline through GrimAge were also significant (p < 0.05), suggesting that epigenetic aging partially mediates these relationships. Sex differences were also observed. Poverty at birth predicted faster epigenetic aging at age 15 for males not females. We also observed that faster epigenetic aging at age 15 was predictive of school discipline of male not female participants at age 22. In contrast, faster epigenetic aging at age 15 was predictive of self-rated health (SRH) of female not male participants at age 22.

**Conclusions:**

This study provides evidence that with some sex differences, race/ethnicity and poverty at birth contribute to accelerated epigenetic aging (GrimAge) by age 15, which in turn predicts poorer self-rated health and increased school discipline issues by age 22. These findings emphasize the importance of early interventions targeting social determinants to mitigate long-term health and behavioral disparities. Addressing these early life conditions is crucial for improving health equity and outcomes in young adulthood.

## 1. Introduction

Socioeconomic factors and race/ethnicity play pivotal roles in determining health outcomes across the lifespan [1–3]. From birth, individuals from marginalized racial and ethnic groups, as well as those born into poverty, face increased exposure to adverse conditions that can affect their health trajectories [4, 5].

Epigenetics, the study of how environmental factors can alter gene expression without changing the DNA sequence, has emerged as a critical field for understanding these long-term impacts [6]. This field provides a framework for investigating how exposures to various social and environmental stressors during critical periods of development can lead to lasting changes in gene expression that influence an individual’s health trajectory [7]. These epigenetic modifications can affect biological processes in ways that predispose individuals to a range of health outcomes, potentially exacerbating disparities linked to socioeconomic status and race/ethnicity [8–12]. By exploring these mechanisms, researchers can gain valuable insights into how early life conditions such as poverty, discrimination, and limited access to resources contribute to health disparities observed later in life [11, 13, 14]. This understanding underscores the complex interplay between genes and the environment, emphasizing that health outcomes are not solely determined by genetic makeup but are profoundly influenced by one’s social context and experiences [15–17]. Within this burgeoning field, GrimAge, an epigenetic clock that predicts lifespan and health span based on DNA methylation patterns, has been identified as a significant biomarker for aging and health prediction [18–21].

Understanding how early life conditions influence GrimAge provides profound insights into the biological mechanisms that link early adversity to later health disparities, highlighting the intersection of social and biological determinants of health. GrimAge, an epigenetic biomarker, measures the biological age of an individual based on DNA methylation patterns, offering a precise tool for assessing the cumulative impact of various environmental exposures. By examining how socioeconomic factors such as poverty and race/ethnicity at birth are associated with changes in GrimAge by adolescence, researchers can elucidate how these early adversities become biologically embedded. This process of biological embedding underscores the ways in which chronic stress, inadequate access to resources, and exposure to environmental toxins, often more prevalent among marginalized groups, can lead to accelerated biological aging. Such insights are crucial for understanding the persistence of health disparities into adulthood, as they reveal that the roots of these disparities extend far beyond immediate social and economic conditions to include long-term biological effects. Consequently, this perspective reinforces the need for comprehensive public health strategies that address both the social determinants of health and their biological consequences, aiming to mitigate the lifelong impact of early adversity and promote health equity.

Despite extensive research on the social determinants of health, there remains a gap in understanding the long-term biological effects of early life socioeconomic and racial/ethnic disparities [22–24]. Particularly, the specific pathways through which poverty and racial/ethnic status at birth influence health and behavior in later life via epigenetic changes are not well elucidated [13, 25, 26]. This study addresses this gap by examining the association between race/ethnicity, poverty at birth, and GrimAge at age 15, and how these epigenetic changes predict self-rated health and school discipline at age 22 [27–29]. Unraveling these mechanisms is crucial for developing targeted interventions to mitigate the adverse effects of early life adversity on long-term health outcomes.

### 1.1. Objectives

The primary objective of this study is to investigate how race/ethnicity and poverty at birth are associated with epigenetic changes at age 15, specifically through the GrimAge biomarker, and to explore how these changes predict self-rated health, depression, school dropout, and school discipline outcomes at age 22. We also explored sex differences in these regards. By focusing on these specific relationships, the study aims to provide a comprehensive understanding of the biological embedding of early life adversity and its implications for health and behavior in young adulthood. This research will contribute to the broader field of health disparities by elucidating the pathways through which social determinants impact long-term health and behavioral outcomes.

## 2. Methods

### 2.1. Design and Setting

The Future of Families and Child Wellbeing Study (FFCWS), formerly known as the Fragile Families and Child Wellbeing Study, is a pioneering research project aimed at understanding the challenges faced by economically disadvantaged families in the United States. The FFCWS follows a birth cohort starting in 1998, tracking children from birth to young adulthood at age 22 in 2022. Detailed information on the study’s sampling techniques and methodologies can be found in previously published literature. This section provides a brief overview of the FFCWS research design.

### 2.2. Ethics

The study protocol was approved by the Institutional Review Board at Princeton University. Informed consent was obtained from all participating families, with parents or legal guardians consenting on behalf of minors, who also provided their assent. All data collection, storage, and analysis procedures were designed to protect participants’ anonymity, and families were compensated for their participation.

### 2.3. Sample and Sampling

The FFCWS recruited a diverse sample of urban families from 20 major U.S. cities, each with a population exceeding 200,000. The study specifically targeted underrepresented families, particularly non-married, Black, and Latino families. Consequently, the study’s sample predominantly consists of low socioeconomic status families, with a substantial representation of Black and Hispanic participants, which does not reflect the overall U.S. population. The analytical sample included 854 families with a Black, Latino, or White families.

### 2.4. Process

Our analysis utilized data from the first and seventh waves of the FFCWS. Socioeconomic position (SEP) data were collected at birth (wave 1, baseline), and outcomes were measured when the offspring were young adults 22 years later (wave 7). The analysis included 733 racial/ethnic diverse families with follow-up data.

### 2.5. Predictors

Baseline data were collected through interviews with both parents, covering parents’ poverty status and family structure at birth. Family poverty status at birth was measured as a continuous measure ranging from 0 to 12.3, with higher number indicator of higher SEP.

### 2.6. Collection of Saliva Sample

During the Year 15 follow-up wave, saliva was collected from the focal children (now teenagers) using Oragene DNA Self-Collection Kits (OGR-500) as described for the year 9 followup with the following modifications. For those who did not complete a home visit, saliva collection kits were sent to participants via mail and after collection participants returned the kits to Westat via FedEx. Participants were discouraged from eating or drinking anything within 30 minutes prior to sample collection. Upon completion of the saliva collection, all participants received $20 [30].

### 2.7. Acquisition and Processing of the DNA Methylation Data

For the DNA methylation analysis, approximately 500 ng of genomic DNA, quantified using the Quant-iT Picogreen dsDNA Assay Kit, underwent bisulfite conversion utilizing the EZ-96 DNA Methylation Kit (Zymo Research). The converted DNA was then analyzed using the Illumina Infinium Human Methylation450K (450K) or the Illumina Infinium MethylationEPIC (EPIC) array, following the manufacturer’s protocols. This process was carried out by the Pennsylvania State College of Medicine Genome Sciences Core facility. To minimize technical variation, DNA samples from ages 9 and 15 were processed concurrently. Samples were randomized to prevent bias. The red and green image pairs were imported into R for analysis. Quality control (QC) of the methylation data was conducted initially using EWAStools. Probes were excluded if their detection values exceeded 0.01 for the 450K array or 0.05 for the EPIC array, or if the number of methylated or unmethylated bead counts was less than four. Probes were also removed based on the ENmix function QCinfo, which was applied with default parameters. Samples were excluded if they had outlier methylation or bisulfite conversion values as identified by the ENmix QC function, or if the predicted sex from the methylation data did not match the recorded sex. Additionally, samples were flagged if sequential samples from the same individual showed genetic discordance between visits. The ENmix preprocessENmix and rcp functions were employed to normalize dye bias, apply background correction, and adjust for probe-type bias [30].

### 2.8. GrimAge Epigenetic Clock

The DNAm GrimAge epigenetic clock was developed by Ake Lu, Steve Horvath and colleagues. The authors used Framingham Heart Study data, including DNA methylation data from the HumanMethylation450K BeadChip array, from 2356 individuals composed of 888 pedigrees to construct a mortality risk estimator from DNA methylation data [31]. First, estimators for twelve plasma proteins and smoking pack years based on blood methylation data were developed. These DNAm estimators, together with chronological age and sex were then regressed on time-to-death (due to all-cause mortality) using an elastic net Cox regression model which selected the following covariates: seven DNAm-based surrogate plasma protein markers (adrenomedullin (*_adm), beta-2-microglobulim (*_B2M), cystatin C (*_Cystatin_C), growth/differentiation factor 15 (GDF-15; *_GDF_15), leptin (*_leptin), plasminogen activator inhibitor type 1 (PAI-1; *_pai_1), tissue inhibitor metalloproteinases 1 (TIMP-1; *_TIMP_1)), DNAm pack-years (*_PACKYRS), chronological age, and sex. The resulting value was transformed to be in the unit of years to generate DNAm GrimAge [31]. AgeAccelGrim is the raw residual resulting from regressing observed GrimAge on chronological age [31]. The authors also examined the inclusion of imputed blood cell composition in their multivariate Cox regression 11 models and demonstrated that AgeAccelGrim remained highly predictive of lifespan and time-to-coronary heart disease. DNAm biomarkers remained predictive of lifespan and time-to-CHD after adjusting for blood cell counts. With the exception of leptin, where inclusion of blood cell counts increased significance, the adjustment generally reduced significance [30].

### 2.9. Statistical Analysis

Data analysis was conducted using STATA version 18.0. Descriptive statistics, including frequencies (percentages) and means (standard deviations), were reported. Bivariate analysis was performed using the Pearson correlation test. For the multivariable analysis, we applied structural equation modeling (SEM) to examine the associations between race/ethnicity, poverty status at birth, youth GrimAge the outcome 15 years later, and depression, school dropout, SRH, and school discipline at age 22. The analysis explored the impact of race/ethnicity on biological aging of the child at age 15 via poverty status at birth. We also ran multi-group model where groups were defined based on sex.

## 3. Results

733 participants entered our analysis. Table 1 shows their descriptive data. 52.80% were Black, 22.51% were Latino, 25.38% were living in married households at baseline, and 47.20% were male.

Bivariate unadjusted correlations are shown in Table 2. As this table shows, GrimAge (methylation) was inversely associated with poverty status, maternal education. being in a married family, and race. Male sex was also associated with higher GrimAge (DNA methylation).

**Figure 1.**
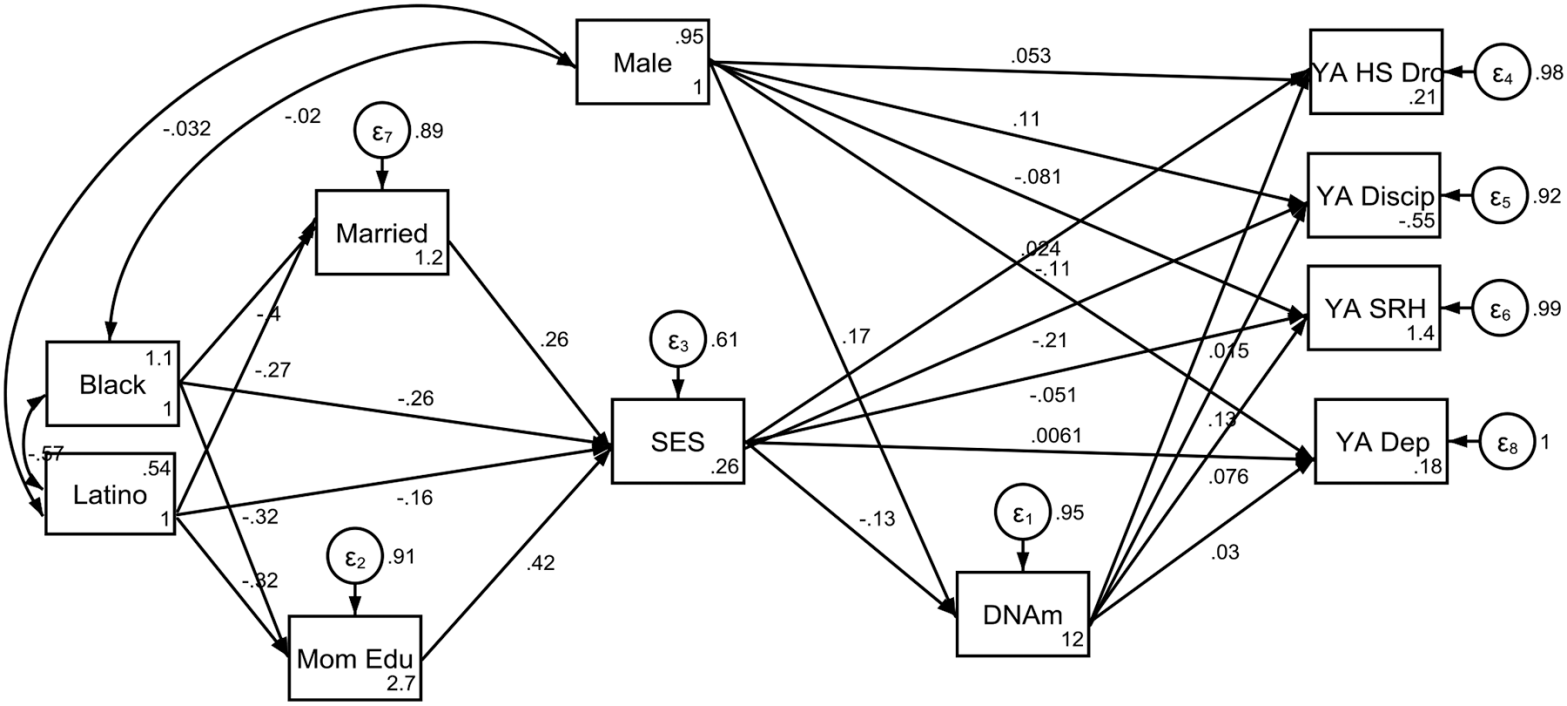
Summary of the Structural Equation Modeling (SEM) Overall

Our findings indicate that Black race and Latino ethnicity were associated with poverty status at birth as well as faster epigenetic aging. Specifically, poverty status at birth partially mediated the effects of race and ethnicity on accelerated aging by age 15. Accelerated aging by age 15 was then predictive of SRH and school discipline at age 22 (Figure 1, Table 3).

**Figure 2.**
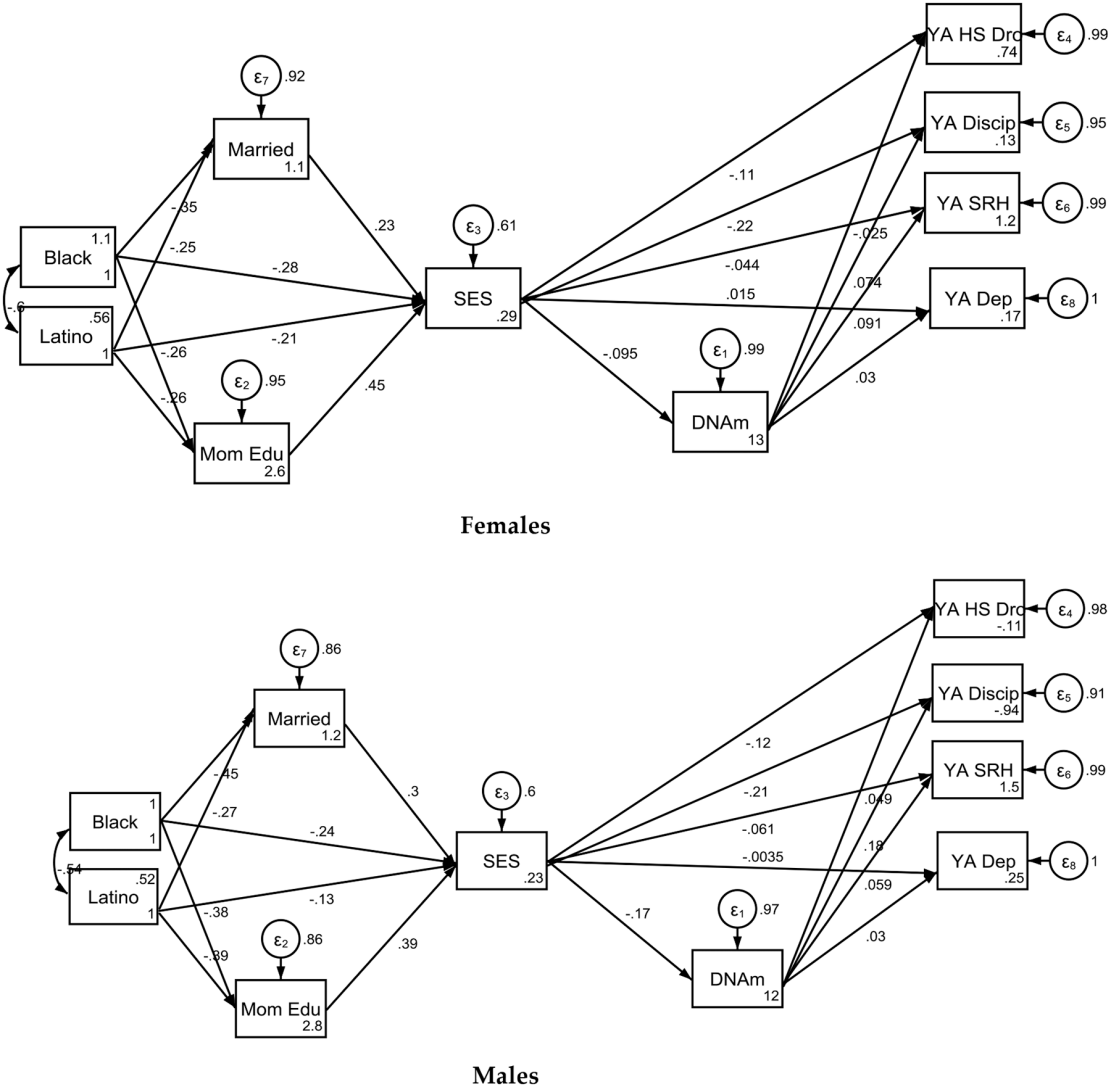
Summary of the Structural Equation Modeling (SEM) by Sex

Our findings indicate that SES at birth was a predictor of faster epigenetic aging of male not female participants in the sample. Specifically, poverty status at birth partially mediated the effects of race and ethnicity on accelerated aging by age 15 for males but not females (Figure 2, Table 4).

We also observed that faster epigenetic aging at age 15 was predictive of school discipline of male not female participants at age 22. In contrast, faster epigenetic aging at age 15 was predictive of SRH of female not male participants at age 22.

## 4. Discussion

The study is guided by two main hypotheses: First, that race/ethnicity and poverty at birth are associated with significant epigenetic changes, as measured by GrimAge, by age 15; and second, that these epigenetic changes serve as predictors of self-rated health, school dropout, depression, and school discipline at age 22. These hypotheses are grounded in existing literature on the social determinants of health and the emerging field of epigenetics, suggesting a biological pathway through which early adversity impacts later life outcomes. By testing these hypotheses, the study aims to advance our understanding of the interplay between social and biological factors in shaping health disparities. We also explored sex differences in these pathways.

This study is anchored in the social determinants of health theory [32–34], which posits that the conditions in which people are born, grow, live, work, and age are critical determinants of health outcomes [35–38]. Epigenetics offers a biological framework for understanding how these social determinants can lead to changes in gene expression that influence health across the lifespan. The GrimAge biomarker, in particular, provides a measurable link between early life adversity and future health risks, allowing for a deeper exploration of the biological embedding of social inequities. This theoretical approach underscores the importance of addressing both social and biological factors in efforts to reduce health disparities.

The findings from this study underscore the long-lasting impact of socioeconomic status (SES) on DNA methylation and cellular aging, as measured by GrimAge, with effects observed 15 years later in children. These epigenetic changes reflect how early life adversity becomes biologically embedded, shaping health and behavioral outcomes over time. GrimAge indicators, which are known to predict mortality risk and aging, are particularly informative in this context. The results suggest that the adversity experienced during early childhood, including the stress of low SES, contributes to accelerated aging at the cellular level. The GrimAge clock’s ability to predict later health outcomes offers a valuable tool for understanding how adversity in childhood impacts biological aging and health later in life.

The GrimAge indicators measured in this study provide a clear link between early life adversity and later health and behavioral problems, including school discipline issues. The accelerated biological aging observed in children from disadvantaged backgrounds appears to manifest not only in physical health disparities but also in behavioral outcomes, such as greater involvement in school discipline problems. The relationship between GrimAge and these outcomes reflects the ways in which stress and adversity during childhood can lead to dysregulation of biological systems, ultimately resulting in both poorer health and greater behavioral challenges. These findings highlight the need for interventions targeting early life stressors to mitigate their long-term impacts on both physical and behavioral health.

Our study also found significant racial and ethnic differences in GrimAge, which warrant further discussion. These disparities are likely rooted in socio-structural factors, including historical and ongoing systemic racism, socioeconomic inequalities, and differential access to resources. For example, Black and Latino children may experience more chronic stress and cumulative adversity, leading to accelerated aging at the cellular level. The historical context of discrimination, structural racism, and residential segregation may exacerbate these biological effects, contributing to the observed racial disparities in GrimAge. These findings emphasize the need to address the broader social determinants of health that disproportionately affect racial and ethnic minorities, as they are clearly linked to biological aging and health disparities later in life.

In addition to racial and ethnic disparities, our study identified sex differences in the impact of early life adversity on health and behavioral outcomes. Specifically, the effects of GrimAge on later outcomes appeared to differ between boys and girls. This raises important questions about the biological and sociological mechanisms underlying these gender/sex differences. From a biological perspective, sex hormones may play a role in mediating the relationship between stress and aging, potentially leading to different trajectories for boys and girls. From a sociological standpoint, gender differences in socialization, stress exposure, and coping strategies may contribute to the observed disparities in health and behavior. Girls and boys may experience different types of stress or have varying support systems, which could influence how early adversity is biologically embedded and later expressed in health and behavior. Understanding these gender/sex differences is crucial for developing targeted interventions that address the specific needs of boys and girls in the context of early adversity.

### 4.1. Implications for Policy and Practice

The findings of this study have significant implications for public health policy and practice. By highlighting the long-term biological impacts of race/ethnicity and poverty at birth, this research underscores the need for early interventions targeting these social determinants to improve health outcomes [39–41]. Policies aimed at reducing poverty and addressing racial/ethnic disparities from birth can potentially mitigate the adverse epigenetic changes associated with GrimAge, leading to better health and behavioral outcomes in later life. Furthermore, this study emphasizes the importance of integrated approaches that combine social and biological perspectives to address health disparities comprehensively.

The study’s key findings reveal a significant association between race/ethnicity, poverty at birth, and GrimAge at age 15, indicating that these early life conditions contribute to accelerated biological aging. Moreover, these epigenetic changes are predictive of poorer self-rated health and higher instances of school discipline at age 22. These results highlight the long-term impact of early socioeconomic and racial/ethnic adversity on both biological and behavioral outcomes. The study provides empirical evidence supporting the hypothesis that early life conditions can lead to epigenetic modifications that influence health and behavior in young adulthood.

The biological plausibility of these findings is rooted in the understanding that early life adversity can lead to chronic stress and inflammation, which in turn can cause epigenetic changes that accelerate aging. The GrimAge biomarker, which incorporates multiple aspects of biological aging, provides a robust measure of these changes. The additive effects of race/ethnicity and poverty on GrimAge suggests that marginalized groups may experience a compounded burden of social and biological adversity. This interpretation underscores the importance of addressing both social inequities and their biological consequences to improve health outcomes for all populations.

The findings of this study align with existing research on the social determinants of health, which consistently demonstrates that early life adversity leads to poorer health outcomes. However, this study adds to the literature by providing a biological mechanism—epigenetic changes as measured by GrimAge—that links these early conditions to later outcomes. Previous studies have shown similar associations between socioeconomic factors, race/ethnicity, and health disparities, but this study’s focus on epigenetics offers a novel perspective on how these disparities are biologically embedded. This comparison highlights the contribution of epigenetics to the broader understanding of health disparities.

The results of this study have significant theoretical implications for the social determinants of health framework. By demonstrating that early life socioeconomic and racial/ethnic conditions can lead to measurable epigenetic changes that predict future health and behavior, this study supports the idea that social adversity is biologically embedded. This finding advances the theoretical understanding of how social factors influence health across the lifespan and underscores the need for a multidisciplinary approach that integrates social and biological perspectives. The study’s contribution to the field of epigenetics also highlights the importance of considering biological mechanisms in theories of health disparities.

### 4.2. Policy Implications

The policy implications of this study are far-reaching. The evidence that early life adversity leads to epigenetic changes that predict poor health and behavioral outcomes suggests that interventions targeting these early conditions could have long-term benefits. Policies aimed at reducing poverty and addressing racial/ethnic disparities from birth could potentially mitigate the adverse effects of these conditions on biological aging and health. Additionally, this study underscores the importance of integrating social and biological approaches in public health strategies to address health disparities comprehensively. Policymakers should consider the findings of this study when designing interventions to improve health equity.

### 4.3. Limitations

While this study provides valuable insights into the long-term impacts of early life adversity, several limitations must be acknowledged. First, the study used data from the Fragile Families and Child Wellbeing Study (FFCWS), which included only a subset of the population, resulting in a sample that was not nationally representative. Additionally, high attrition rates due to the long-term follow-up further limit the generalizability of the findings. Another significant limitation is that data on fathers was not collected after the child reached age 9, which may have omitted crucial paternal influences on health and development.

Measurement errors are also a concern, particularly in the assessment of biomarkers like GrimAge and self-rated health, which could introduce bias into the results. Furthermore, the reliance on self-reported data for health and behavioral outcomes may have introduced reporting bias, potentially skewing the associations observed. The length of follow-up also poses challenges, as attrition over time could lead to selection bias, with those who remained in the study potentially differing in important ways from those who were lost to follow-up.

The generalizability of the results is limited by the specific population studied, which may not reflect broader or more diverse populations. This raises the need for future research to replicate and validate these findings in other groups. Finally, sample selection and measurement error related to long-term data collection and the specific measures used in this study should be carefully considered when interpreting the results, as they may impact the observed associations.

Despite these limitations, this study offers important evidence on the biological embedding of social adversity and its long-term consequences on health outcomes, contributing to our understanding of how early life experiences shape adult health trajectories.

### 4.4. Future Research Directions

Future research should build on the findings of this study by exploring additional mechanisms linking early life adversity to health outcomes. Longitudinal studies that follow individuals from birth to adulthood are needed to validate the observed associations and further elucidate the pathways involved. Additionally, research should investigate the potential for interventions to reverse or mitigate the adverse epigenetic changes associated with early life adversity. Understanding the role of other epigenetic markers and exploring the interaction between genetic and environmental factors will also be important for advancing the field. Future studies should aim to replicate these findings in diverse populations to enhance their generalizability.

## 5. Conclusion

In conclusion, this study provides compelling evidence that race/ethnicity and poverty at birth are associated with significant epigenetic changes, as measured by GrimAge, at age 15, and that these changes predict poorer self-rated health and higher instances of school discipline at age 22. These findings highlight the long-term impact of early life adversity on both biological and behavioral outcomes and underscore the importance of addressing social determinants from birth to improve health equity. By integrating social and biological perspectives, this study contributes to a deeper understanding of health disparities and provides a foundation for developing targeted interventions to reduce these disparities.

## Figures and Tables

**Table 1.** shows descriptive data (n = 733).

	N	%
Race		
White	346	47.20
Black	387	52.80
Ethnicity		
Non-Latino	568	77.49
Latino	165	22.51
Household Marital Status		
Not Married	547	74.62
Married	186	25.38
Sex		
Female	387	52.80
Male	346	47.20

**Table 2.** Bivariate Correlations (Pearson Correlation)

	1	2	3	4	5	6	7	8	9	10	11
1 Black	1.00										
2 Latino	−0.56	1.00									
3 Maternal Education (1−4)	−0.15	−0.13	1.00								
4 Married	−0.27	−0.04	0.39	1.00							
5 Income to Poverty Ratio	−0.28	−0.09	0.57	0.49	1.00						
6 Male	−0.04	0.00	0.01	0.07	0.00	1.00					
7 Young Adult Depression	−0.09	0.05	−0.08	−0.06	0.00	0.03	1.00				
8 Young Adult SRH	−0.04	0.04	−0.05	−0.07	−0.07	−0.06	0.09	1.00			
9 Young Adult School Discipline	0.29	−0.10	−0.23	−0.26	−0.24	0.11	0.07	0.00	1.00		
10 School Drop Out	0.09	−0.02	−0.18	−0.09	−0.14	0.07	0.05	−0.03	0.25	1.00	
11 DNAm Grim	0.14	−0.01	−0.11	−0.08	−0.12	0.17	0.04	0.05	0.15	0.05	1.00

All correlation coefficients larger than 0.03 are statistically significant at p < 0.05

**Table 3.** Summary of the Structural Equation Modeling (SEM) in the Pooled Sample

	Coefficient	std. err.	95%	CI	*p*
Structural					
Maternal Education at Baseline					
Ethnicity (Latino)	−0.32	0.04	−0.40	−0.24	< 0.001
Race (Black)	−0.32	0.04	−0.40	−0.24	< 0.001
Intercept	2.67	0.08	2.51	2.82	< 0.001
Poverty Ratio (Baseline)					
Maternal Education (Baseline)	0.42	0.03	0.36	0.48	< 0.001
Married Household	0.26	0.03	0.20	0.32	< 0.001
Ethnicity (Latino)	−0.16	0.04	−0.24	−0.09	< 0.001
Race (Black)	−0.26	0.04	−0.33	−0.18	< 0.001
Intercept	0.26	0.10	0.06	0.46	0.010
Married Household					
Ethnicity (Latino)	−0.27	0.04	−0.35	−0.19	< 0.001
Race (Black)	−0.40	0.04	−0.48	−0.33	< 0.001
Intercept	1.16	0.06	1.03	1.28	< 0.001
Grim Age (Wave 6)					
Poverty Ratio (Baseline)	−0.13	0.03	−0.20	−0.06	< 0.001
Male Child	0.17	0.04	0.10	0.24	0.000
Intercept	12.38	0.33	11.74	13.03	0.000
High School Drop Out (Wave 7)					
Poverty Ratio (Baseline)	−0.11	0.04	−0.18	−0.04	0.001
Grim Age (Wave 6)	0.01	0.04	−0.06	0.09	0.689
Male Child	0.05	0.04	−0.02	0.13	0.153
Intercept	0.21	0.47	−0.71	1.13	0.658
Any School Discipline (Wave 7)					
Poverty Ratio (Baseline)	−0.21	0.03	−0.28	−0.14	< 0.001
Grim Age (Wave 6)	0.13	0.04	0.06	0.20	< 0.001
Male Child	0.11	0.04	0.04	0.18	0.003
Intercept	−0.55	0.45	−1.43	0.34	0.224
YA SRH (Wave 7)					
Poverty Ratio (Baseline)	−0.05	0.04	−0.12	0.02	0.152
Grim Age (Wave 6)	0.08	0.04	0.00	0.15	0.042
Male Child	−0.08	0.04	−0.15	−0.01	0.029
Intercept	1.42	0.47	0.49	2.35	0.003
YA MDD (Wave 7)					
Poverty Ratio (Baseline)	0.01	0.04	−0.07	0.08	0.872
Grim Age (Wave 6)	0.03	0.04	−0.05	0.11	0.448
Male Child	0.02	0.04	−0.05	0.10	0.549
Intercept	0.18	0.50	−0.79	1.16	0.711

MDD: Major Depressive Disorder, SRH: Self-Rated Health, YA: Young Adult.

**Table 4.** Summary of Structural the Equation Modeling (SEM) by Sex

	Coefficient	std. err.	95%	CI	*p*
**Female**					
Maternal Education at Baseline					
Ethnicity (Latino)	−0.26	0.06	−0.38	−0.14	< 0.001
Race (Black)	−0.26	0.06	−0.37	−0.14	< 0.001
Intercept	2.55	0.12	2.32	2.79	< 0.001
Poverty Ratio (Baseline)					
Maternal Education (Baseline)	0.45	0.04	0.37	0.53	< 0.001
Married Household	0.23	0.04	0.14	0.31	< 0.001
Ethnicity (Latino)	−0.21	0.05	−0.31	−0.11	< 0.001
Race (Black)	−0.28	0.05	−0.38	−0.18	< 0.001
Intercept	0.29	0.14	0.02	0.56	0.033
Married Household					
Ethnicity (Latino)	−0.25	0.06	−0.36	−0.13	< 0.001
Race (Black)	−0.35	0.06	−0.47	−0.24	< 0.001
Intercept	1.05	0.10	0.86	1.24	< 0.001
Grim Age (Wave 6)					
Poverty Ratio (Baseline)	−0.10	0.05	−0.19	0.00	0.051
Intercept	13.18	0.47	12.25	14.10	< 0.001
High School Drop Out (Wave 7)					
Poverty Ratio (Baseline)	−0.11	0.05	−0.21	−0.01	0.025
Grim Age (Wave 6)	−0.02	0.05	−0.12	0.07	0.626
Intercept	0.74	0.67	−0.58	2.05	0.275
High School Discipline (Wave 7)					
Poverty Ratio (Baseline)	−0.22	0.05	−0.31	−0.12	< 0.001
Grim Age (Wave 6)	0.07	0.05	−0.02	0.17	0.136
Intercept	0.13	0.66	−1.16	1.42	0.844
YA SRH (Wave 7)					
Poverty Ratio (Baseline)	−0.04	0.05	−0.14	0.05	0.368
Grim Age (Wave 6)	0.09	0.05	−0.01	0.19	0.073
Intercept	1.19	0.68	−0.14	2.52	0.080
YA MDD (Wave 7)					
Poverty Ratio (Baseline)	0.01	0.05	−0.09	0.12	0.775
Grim Age (Wave 6)	0.03	0.05	−0.08	0.14	0.584
Intercept	0.17	0.72	−1.24	1.58	0.815
**Male**					
Maternal Education at Baseline					
Ethnicity (Latino)	−0.39	0.06	−0.50	−0.28	< 0.001
Race (Black)	−0.38	0.06	−0.49	−0.27	< 0.001
Intercept	2.77	0.11	2.55	2.98	< 0.001
Poverty Ratio (Baseline)					
Maternal Education (Baseline)	0.39	0.05	0.30	0.48	< 0.001
Married Household	0.30	0.05	0.21	0.39	< 0.001
Ethnicity (Latino)	−0.13	0.05	−0.23	−0.02	0.015
Race (Black)	−0.24	0.05	−0.34	−0.13	< 0.001
Intercept	0.23	0.15	−0.06	0.53	0.117
Married Household					
Ethnicity (Latino)	−0.27	0.06	−0.39	−0.16	< 0.001
Race (Black)	−0.45	0.05	−0.56	−0.34	< 0.001
Intercept	1.24	0.09	1.07	1.41	< 0.001
Grim Age (Wave 6)					
Poverty Ratio (Baseline)	−0.17	0.05	−0.27	−0.07	0.001
Intercept	12.31	0.46	11.40	13.21	< 0.001
High School Drop Out (Wave 7)					
Poverty Ratio (Baseline)	−0.12	0.05	−0.22	−0.01	0.027
Grim Age (Wave 6)	0.05	0.05	−0.06	0.15	0.367
Intercept	−0.11	0.67	−1.43	1.20	0.864
High School Discipline (Wave 7)					
Poverty Ratio (Baseline)	−0.21	0.05	−0.30	−0.11	< 0.001
Grim Age (Wave 6)	0.18	0.05	0.08	0.28	< 0.001
Intercept	−0.94	0.64	−2.19	0.32	0.144
YA SRH (Wave 7)					
Poverty Ratio (Baseline)	−0.06	0.05	−0.16	0.04	0.250
Grim Age (Wave 6)	0.06	0.05	−0.05	0.17	0.273
Intercept	1.50	0.68	0.17	2.83	0.027
YA MDD (Wave 7)					
Poverty Ratio (Baseline)	0.00	0.06	−0.11	0.11	0.950
Grim Age (Wave 6)	0.03	0.06	−0.08	0.14	0.606
Intercept	0.25	0.71	−1.14	1.65	0.720

MDD: Major Depressive Disorder, SRH: Self-Rated Health, YA: Young Adult.

## Data Availability

FFCWS data are available to public at Office of Population Research data repository available at https://oprdata.princeton.edu/archive/restricted/Default.aspx.
